# ECMO in nonintubated patients as a bridge to lung transplant: our experience

**DOI:** 10.1186/cc10704

**Published:** 2012-03-20

**Authors:** M Chierichetti, A Santini, F Pagan, S Crotti, A Lissoni, L Gattinoni

**Affiliations:** 1 Fondazione IRCCS Ca' Granda Ospedale Maggiore Policlinico Milano, Milan, Italy

## Introduction

Extracorporeal membrane oxygenation (ECMO) has now been used by an expanding number of centres for bridging to lung transplant (LTx) in patients with advanced cardiac and respiratory failure [1]. ECMO has been used for bridging to LTx almost exclusively in patients receiving mechanical ventilation. In order to avoid the drawbacks and complications associated with intubation and prolonged mechanical ventilation we hypothesized that the use of venovenous ECMO (VV-ECMO) in awake and spontaneously breathing patients might be an option for respiratory support in those patients who are severely deteriorating while waiting for lung transplant.

## Methods

We performed a retrospective analysis of seven patients (three female, mean age 31.7 ± 12.1 years) who underwent lung transplant while on ECMO support between May 2009 and October 2011 and who had not been ventilated for more than 24 hours before the LTx. All patients were fully awake and they kept on receiving noninvasive ventilation for a variable amount of time per day after ECMO support was started, according to clinical evaluation. Mean blood gas values before ECMO support was started were: pH 7.26 ± 0.13, PaCO_2 _81.7 ± 31.6, PaO_2 _151.4 ± 164.2, PEEP 9 ± 4, FiO_2 _83 ± 20, mean time on ECMO before LTx 11.7 ± 17.7 days.

## Results

All patients survived successfully until the transplant. All patients underwent BLTx on VV-ECMO support, three were converted to VA during transplant and then back to VV at the end of the procedure. One patient died after BLTx due to hemorrhagic complications. Mean ECMO support was BloodFlow 3.1 ± 0.8 l/minute, GasFlow 4.7 ± 2.5 l/minute, no one needed mechanical ventilation before BLTx. After lung transplant five patients remained intubated and they were ventilated for 13.9 ± 16.4 days. Mean duration of ECMO support after LTx was 4.7 ± 5.4 days. Mean ICU LOS after LTx was 18 ± 17.9 days. Among this population three patients developed hemorrhagic complication, two primary graft dysfunction, two neuromuscular dysfunction, while only one chronic renal failure.

## Conclusion

Our experience shows that bridge to lung transplant with VV-ECMO in awake and spontaneously breathing patients is not only feasible but also successful. Survival to BLTx in our center was 100%, while survival after BLTx is comparable to that of patients who were not on ECMO support.
